# Development and management of systemic lupus erythematosus in an HIV-infected man with hepatitis C and B co-infection following interferon therapy: a case report

**DOI:** 10.4076/1752-1947-3-7289

**Published:** 2009-06-10

**Authors:** Iain J Abbott, Christina C Chang, Matthew J Skinner, Alison Street, Greg Perry, Catriona McLean, Edwina J Wright, Paul U Cameron

**Affiliations:** 1 Department of Infectious Diseases, Alfred Hospital, Melbourne, Victoria, 3004 Australia; 2 Department of Immunology, Alfred Hospital, Melbourne, Victoria, 3004 Australia; 3 Department of Haematology, Alfred Hospital, Melbourne, Victoria, 3004 Australia; 4 Department of Nephrology, Alfred Hospital, Melbourne, Victoria, 3004 Australia; 5 Department of Anatomical Pathology, Alfred Hospital, Melbourne, Victoria, 3004 Australia; 6 Department of Medicine, Monash University, Melbourne, Victoria, 3004 Australia; 7 Burnet Institute, Melbourne, Victoria, 3004 Australia

## Abstract

**Introduction:**

The association of human immunodeficiency virus and immune dysfunction leading to development of autoimmune markers is well described, but human immunodeficiency virus infection is relatively protective for the development of systemic lupus erythematosus. In contrast, development of systemic lupus erythematosus with hepatitis C and with interferon therapy is well described in a number of case reports. We here describe the first case of systemic lupus erythematosus developing in a man infected with human immunodeficiency virus, hepatitis C and hepatitis B co-infection where the onset seems to have been temporally related to interferon therapy.

**Case presentation:**

We report the occurrence of systemic lupus erythematosus complicating interferon-α therapy for hepatitis C in a 47-year-old asplenic male with haemophilia co-infected with human immunodeficiency virus and hepatitis B. He presented with a truncal rash, abdominal pains and headache and later developed grade IV lupus nephritis requiring haemodialysis, mycophenolate mofetil and steroid therapy. We were able to successfully withdraw dialysis and mycophenolate while maintaining stable renal function.

**Conclusion:**

Interferon-α is critical in antiviral immunity against hepatitis C but also acts as a pathogenic mediator for systemic lupus erythematosus, a condition associated with activation of plasmacytoid dendritic cells that are depleted in human immunodeficiency virus infection. The occurrence of auto-antibodies and lupus-like features in the coinfections with hepatitis C require careful assessment. Immunosuppressant therapy for lupus risks exacerbating underlying infections in patients with concurrent human immunodeficiency virus, hepatitis B and C.

## Introduction

Co-infection with hepatitis C (HCV) and human immunodeficiency virus (HIV) is a common problem of increasing clinical significance. Interferon (INF) therapy is the mainstay of HCV treatment. HCV and HIV have been associated with the development of autoimmune markers and disease; INF therapy compounds this risk.

## Case presentation

A 47-year-old Caucasian man presented in May 2006 with abdominal pain, headache for six weeks an undiagnosed truncal rash for eight months with a background of haemophilia A (5% factor VIII activity), HIV, Genotype 1b HCV and HBV coinfection. His HIV was well-controlled on lamivudine, tenofovir and ritonavir-boosted lopinavir; his cluster of differentiation antigen 4+ (CD4+) T-cell count was 700cells/µL (28%) and he had an undetectable HIV RNA. He had no prior Acquired Immune Deficiency Syndrome (AIDS)-defining illnesses. He had compensated liver cirrhosis (Child Pugh class A, grade 2 inflammation, stage 4 fibrosis) and had previously failed to achieve HCV suppression after 19 weeks of pegylated IFN (PEG-IFN) and ribavarin therapy from March to August 2005. Other comorbidities include prior traumatic splenectomy in February 2004, osteoporosis, renal calculi, inactive psoriasis and mild obstructive sleep apnoea.

On presentation, he was hypertensive at 200/100 mmHg without fundoscopic or focal neurological changes. There were no peripheral stigmata of chronic liver disease. Investigations on presentation demonstrated new, mildly increased creatinine 0·13 mmol/L (normal range [NR] 0·06-0·11) but with marked proteinuria 8·79 g/day (NR<1·5), and a reduced creatinine clearance of 0·94 ml/sec (NR 1·50-2·50) with dysmorphic red blood cells on urinalysis. Full blood examination was normal with a haemoglobin level of 136 g/L, white blood cell count 8·39 × 10^9^/L and platelets 173 × 10^9^/L. Erythrocyte sedimentation rate was 103 (NR 1-10), C-reactive peptide 10 (NR 0-5), liver function test showed a low albumin 18 (NR 35-52), normal bilirubin 16 µmol/L (NR <21) and ALT 26 U/L (NR 0-40), and a slightly raised GGT 83 U/L (NR 12-64) and ALP 209 U/L (NR <110). The computer tomography (CT) brain scan was normal, but magnetic resonance imaging (MRI) showed increased deep white matter hyperintensities. The electrocardiogram and echocardiogram suggested left ventricular hypertrophy with normal systolic function. Antineutrophil cytoplasmic antibodies (ANCA), myeloperoxidase and proteinase-3 antibodies, cryoglobulins, serum protein electrophoresis and urine Bence Jones proteins were negative. A CT scan of the abdomen revealed thickened terminal ileum and moderate ascites. Diagnostic paracentesis revealed a serum-ascites-albumin gradient of more than 12 which was non-infective.

Meloxicam and tenofovir were ceased because of worsening renal function and zidovudine was instituted in place. Perindopril was commenced at 2 mg, 4 mg then 8 mg and, later, together with 10mg amlodipine, 12·5 mg hydrocholorothiazide and 0·5 mg prazosin twice daily for control of resistant hypertension.

**Figure 1:**
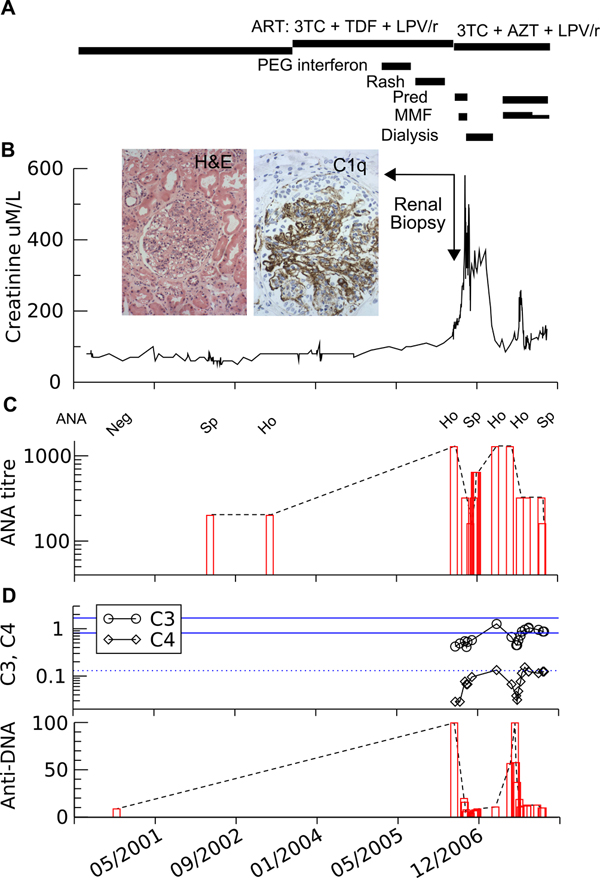
**Patient's clinical course**. **A:** summary of therapy. **B:** serum creatinine. **Insert:** renal biopsy, haematoxylin and eosin stain (magnification ×200) and C1q immunoperoxidase stain (magnification ×400), showing diffuse active lupus nephritis class IV. Highlighting granular deposits in mesangium and capillary walls of all immunoglobulins and complements ("full house immunofluorescence"), distinguishing it from primary membranoproliferative glomerulonephritis. **C:** changes in autoantibodies pattern (Ho, homogenous; Sp, speckled) and **D:** complement C3 and C4 (lower limit of normal for C4 and ref range for C3 are shown). Changes in anti-DNA antibodies with time. Pred, prednisolone; MMF, mycophenolate mofetil.

Antinuclear antibody (ANA), which had formerly been negative five years prior and weakly positive in 2004 (Figure 1C), was now strongly positive (>1:1280, homogeneous) in association with elevated anti-double-stranded DNA (dsDNA) antibodies (>100), and hypocomplementemia (C3 0·44 and C4 0·03) consistent with active systemic lupus erythematosus (SLE). The previous skin biopsy of the truncal rash, originally thought to be secondary to a macrolide antibiotic drug reaction, was reviewed and showed a lichenoid reaction involving hair follicles without eosinophils, also suggestive of SLE. Renal biopsy was considered but deferred because of risks associated with haemophilia. Prednisolone 37·5 mg daily (0·5 mg/kg, dose adjusted for ritonavir coadministration) was empirically commenced on 2 June 2006 for treatment of lupus nephritis.

Further deterioration in renal function to creatinine 0·17 mmol/L and lack of clinical improvement led to a renal biopsy on 16 June 2006 which revealed class IV lupus nephritis (Figure 1). Mycophenolate mofetil 1g twice daily was added on 21 June with clinical improvement and the patient was discharged home on 17 July.

He was readmitted one week later with worsening renal impairment creatinine 0·26 mmol/L, lethargy, anaemia and hypotension necessitating cessation of antihypertensives. Pulse methylprednisolone, 500 mg daily for three days, and intravenous immunoglobulin, 30 g daily, was started for further renal deterioration. Despite this, urgent haemodialysis was required on 27 July for marked acidosis, oliguria and creatinine 0·54 mmol/L. Immunosuppression ceased at this time. Further complications included microangiopathic haemolysis, steroid myopathy, atrial flutter and spontaneous bacterial peritonitis.

After five months of thrice-weekly haemodialysis, a dramatic improvement in the patient's renal function and proteinuria allowed the successful cessation of haemodialysis on 26 December 2006.

In May 2007, a gradual rise in ANA, anti-DNA levels and creatinine were noted necessitating the reinstitution of mycophenolate 1 g twice daily and prednisolone 40 mg daily (Figure 1B). Entecavir 1 mg was commenced in 20 July 2007 for hepatitis B virus (HBV) treatment.

In November 2007, while on 20 mg prednisolone and mycophenolate 25 mg twice daily, Coombs-negative haemolytic anaemia developed, rendering the patient transfusion-dependent, which improved with darbopoeitin-alfa 80 µg weekly. Persistent re-accumulation of ascites with liver decompensation led to repeated paracentesis every 2-3 weeks, complicated by further episodes of peritonitis. His HCV viral load remains high at 2 million IU but HBV and HIV viral loads remain undetectable with a CD4 count of 274 (13%). His current creatinine is maintained between 0·13 and 0·18 mmol/L and prednisolone has been weaned to 17·5 mg daily and mycophenolate has been ceased.

## Discussion

The occurrence of SLE in the setting of HCV and INF administration is now well established [1]-[3] and represents an accentuation of the underlying cytokine changes described in SLE pathogenesis [4,5]. Development of SLE is favoured by both reduced tumour necrosis factor (TNF) α following anti-TNF therapy or by increased IFNα. In coinfected patients, the risk of development of SLE associated with INFα HCV therapy, HCV is significantly reduced by the concomitant HIV infection [6].

In HIV, the risk of developing autoantibodies and autoimmune disease depends on the time in the disease course and may be stratified by HIV manifestations, CD4 count and HIV viraemia [6]. Autoimmune diseases predominates in stage 1 (acute HIV) in the setting of relatively intact host immunity and immune activation, and in stage 4 with restoration of immune function with anti-retroviral therapy (ART). The susceptibility of CD4+ CD25+ regulatory T cells to HIV infection [7] and CD4 loss may both contribute to dysregulation of immune responses and favour autoimmunity. HIV can increase autoantibodies through non-specific stimulation of B-cells [8] and viral and mammalian DNA can trigger toll-like receptor (TLR) ligation and IFN production by plasmacytoid dendritic cells (pDC) [9].

HCV is a potential aetiological factor for SLE and alone can mimic SLE both clinically and serologically; the presence of highly specific autoantibodies for SLE such as anti-Smith, anti-dsDNA and anti-nucleosome antibodies, distinguishes SLE [2]. In our case, the presence of anti-dsDNA antibodies, hypocomplementemia and the histological diagnosis of class IV lupus nephritis established the diagnosis.

PEG-IFN is commonly associated with development of autoantibodies and with autoimmune disorders in 4% to 19% of patients, and SLE-like syndromes have been reported in 0.15% to 0.7% of patients on INF therapy [1], with the onset ranging from one month to seven years after INF administration. Most present with arthralgia and a highly elevated ANA.

Changes in type I IFN levels and pDC redistribution and activation are important in pathogenesis of SLE [3]. IFN-α is an autocrine survival factor for pDC that are potent IFN-producing cells and can activate immature dendritic cells to increase presentation of self-antigens to autoreactive T and B cells [10]. In response to viral infection, pDC further increase IFN production via TLR7 and TLR9 pathways [11], leading to increased pDC migration and maturation [12].

**Figure 2:**
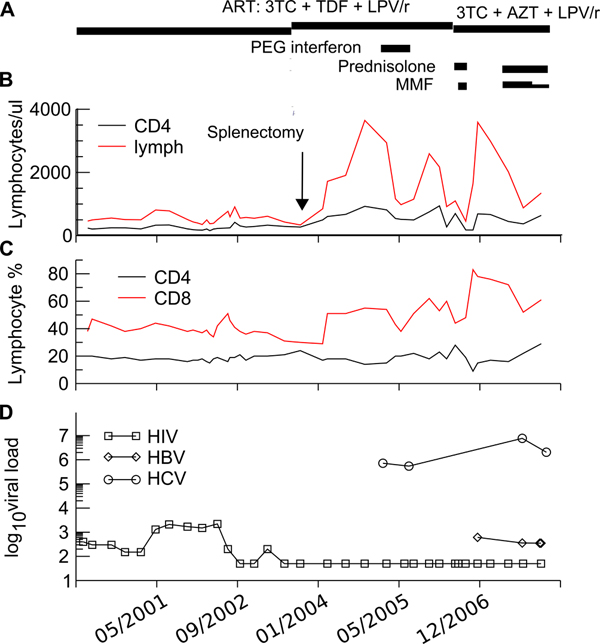
**Changes in viral load and lymphocytes pre and post therapy**. **A:** summary of therapy. **B:** lymphocyte subset numbers and **C:** lymphocyte proportion during ART and in response to splenectomy and anti-retroviral therapy, Interferon-α therapy and immunosuppression. **D:** Changes in viral load of human immunodeficiency virus, hepatitis C and hepatitis B during therapy.

In this case, IFN therapy was most likely the critical factor leading to SLE and was temporally related to the development of the skin rash and evolution of SLE as demonstrated in Figure 2. HIV infection is protective for development of SLE [6], and SLE is rare in HCV/HIV coinfection compared to HCV alone. Depletion of INF-producing pDCs during HIV infection [13] may contribute to this protective effect of HIV. DCs can be infected by HIV, and TLR9 signalling-changes in pDC function may be reduced indirectly by HIV gp120 [14]. Loss of pDC in HIV infection usually parallels the loss of CD4 [15], but the rate of recovery of DC and CD4 T cells may be independent.

What factors may have been important in the development of SLE in this patient at this time? SLE-immune reconstitution disease was considered; however, there was no significant change in HIV RNA or CD4+ T-cell count to precipitate the disease onset, and his ART regime had been unaltered for more than five years. His peripheral CD4 count (Figure 2) had increased as expected in absolute numbers following splenectomy in 2004, but CD4 percentage was stable. Normalised pDC numbers expected with prolonged ART [16,17] may have been permissive for the IFN-induced development of SLE.

A unique feature of this case was the initial recovery of renal function despite biopsy-proven class IV SLE nephritis, allowing cessation of dialysis and immunosuppressants. The subsequent decline in renal function with SLE flare raised the inevitable and vexed issue of further immunosuppressants; choice, timing and dose. Immunosuppressant therapy is a double-edged sword in this patient with known HIV, HBV and HCV coinfection; his chronic liver disease only adds to his already significant infective risk. Immunosuppressant therapy has been associated with reduced control of HIV [15] and has been clearly associated with HBV-related flares, reactivation and mortality. In this case, the HBV viral loads were well controlled with tenofovir and subsequent switch to entecavir. The progression of HCV-related liver disease with immunosuppressants remains unclear, and his viral load remains high.

In view of his known liver disease and anaemia, we avoided azathioprine and used mycophenolate and prednisolone at small ritonavir-boosted doses. The patient remains on steroid therapy and maintains stable renal function three months post-cessation of mycophenolate with reduction in autoantibody levels.

## Conclusion

This case report illustrates the complex interactions between HCV, HIV and SLE and antiviral immunity mediated by IFNα and pDC. INF therapy is a critical component of current HCV treatment and its risk including development of autoantibodies, and autoimmune diseases needs to be balanced against its benefits. The development of autoantibodies and lupus-like features in patients with chronic HCV needed careful assessment and monitoring. The role of immunosuppressants for treatment of autoimmune diseases in the setting of concomitant HIV, HCV and HBV coinfection is difficult. Further research into the complex interplay between immunomodulators and ART in the presence of autoimmune diseases and viral hepatides is necessary.

## Abbreviations

AIDS: Acquired Immune Deficiency Syndrome; ANCA: antineutrophil cytoplasmic antibodies; ANA: antinuclear antibody; ART: anti-retroviral therapy; CD: cluster of differentiation antigen; CT: computed tomography; dsDNA: double-stranded DNA; HBV: hepatitis B virus; HCV: hepatitis C; HIV: human immunodeficiency virus; IFN: interferon; MRI: magnetic resonance imaging; NR: normal range; pDC: plasmacytoid dendritic cell(s); PEG-IFN: pegylated-interferon; SLE: systemic lupus erythematosus; TNF: tumour necrosis factor; TLR: toll-like receptor.

## Consent

Written informed consent was obtained from the patient for publication of this case report and accompanying images. A copy of the written consent is available for review by the Editor-in-Chief of this journal.

## Competing interests

MJS received a travel grant from Gilead in 2005 and 2006. All remaining authors: no conflicts.

## Authors' contributions

IJA, CCC and PUC were responsible for writing the manuscript. MJS, AS, GP and EJW provided clinical details and contributed to the final manuscript. CM examined the biopsies, provided pathological details and micrographs and revised the manuscript. All authors read and approved the final manuscript.
